# Retrospective Memories of Parents about Behaviour of Their Children During Lockdown

**DOI:** 10.1192/j.eurpsy.2022.1318

**Published:** 2022-09-01

**Authors:** N. Burlakova, F. Diusimbeeva

**Affiliations:** Lomonosov Moscow State University, Faculty of Psychology, Department Of Neuro- And Pathopsychology, Moscow, Russian Federation

**Keywords:** parental memories, children during lockdown, Covid-19

## Abstract

**Introduction:**

The trace in the memory left by the COVID-19 pandemic is no less important than the immediate reaction on it.

**Objectives:**

The objective was to study the parent-child relations during the strict lockdown (April 2020) on the material of parental memories focusing on emotional reactions and behaviour of children.

**Methods:**

The material was collected in July-August 2020 in a small city in south Russia. The group included 88 parents (average age 34±5). 42 parents had an only child, 38 two children and 7 from 3 to 5 children. Methods used in the study included questionary, half-structured interview.

**Results:**

61% estimated the lockdown as “very stressful and difficult.” Vast majority (86 parents) said that the situation was constantly discussed in the family, 2 parents demonstrated the reaction of denial saying that the situation was never discussed at home. Respondents mentioned “increase of anxiety,” “insecurity,” “conflicts within the family,” etc. In most cases, parents did not succeed to provide a constructive and balancing explanation, which would answer the child’s wish to understand the situation. Describing the children’s reactions on the changes in the usual way of life, parents stressed anxious (15%) and explosive-angry reactions (10%), with prevalence of apathy, tendency to “stick to” the adult (45%). 30% of parents did not notice any changes in their children’s behaviour.

**Conclusions:**

Results demonstrate the need in clinical-psychological solutions, which would be designed for wide use (“collective patient”) and aimed at explanation of the situation of pandemic to the children of different ages, belonging to different social groups.

**Disclosure:**

No significant relationships.

